# A Global Survey of Self-Reported Cancer Screening Practices by Health Professionals for Kidney Transplant Candidates and Recipients

**DOI:** 10.3389/ti.2024.13965

**Published:** 2025-01-20

**Authors:** Nida Saleem, Wai H. Lim, Jacqueline H. Stephens, Annabelle Wilson, Billie Bonevski, Allison Jaure, Armando Teixeira-Pinto, Eleonora Dal Grande, Martin Howell, Farzaneh Boroumand, Anita van Zwieten, Chandana Guha, Nicole Scholes-Robertson, Steven J. Chadban, Carmel M. Hawley, Jonathan C. Craig, Jeremy R. Chapman, Danyal Hassan, Greg Knoll, Naoka Murakami, Germaine Wong

**Affiliations:** ^1^ Flinders Health and Medical Research Institute, College of Medicine and Public Health, Flinders University, Bedford Park, SA, Australia; ^2^ Centre for Kidney Research, Kids Research Institute, The Children’s Hospital at Westmead, Westmead, NSW, Australia; ^3^ Department of Renal and Transplantation Medicine, Westmead Hospital, Westmead, NSW, Australia; ^4^ Department of Renal Medicine, Sir Charles Gairdner Hospital, Perth, WA, Australia; ^5^ Faculty of Health and Medical Science, University of Western Australia, Perth, WA, Australia; ^6^ Sydney School of Public Health, University of Sydney, Sydney, NSW, Australia; ^7^ Department of Renal Medicine, Kidney Centre, Royal Prince Alfred Hospital, Sydney, NSW, Australia; ^8^ Department of Nephrology, University of Queensland, Brisbane, QLD, Australia; ^9^ Department of Nephrology, Princess Alexandra Hospital, Brisbane, QLD, Australia; ^10^ Department of Renal and Transplantation Medicine, Shifa International Hospital, Islamabad, Pakistan; ^11^ Division of Nephrology, Department of Medicine, The University of Ottawa, Ottawa, ON, Canada; ^12^ Division of Nephrology, Kidney Research Centre, The Ottawa Hospital Research Institute, Ottawa, ON, Canada; ^13^ Division of Nephrology, Washington University in St. Louis, St. Louis, MO, United States

**Keywords:** kidney transplantation, cancer screening, transplant candidates, transplant recipients, cancer

## Abstract

Cancer is a major cause of morbidity and mortality in kidney transplant recipients. Health professionals have a critical role in promoting cancer screening participation. From March 2023 to February 2024, an online survey was distributed to kidney transplant health professionals globally to assess their screening practices. We compared their reported screening practices to recommended guidelines and analyzed factors associated with these practices. We received 97 responses, and most were nephrologists (70%), and around 80% recommended breast, colorectal, and cervical cancer screening for kidney transplant candidates and recipients. About 85% recommended lung cancer screening for higher-risk individuals. Skin cancer screening recommendations varied from 69% for transplant candidates and 84% for recipients. Self-reported cervical cancer screening practices were most concordant with recommended guidelines, followed by breast and skin cancers. Barriers reported included a lack of cancer screening awareness (28%), perceived financial constraints (35%), and deficient structured cancer screening systems (51%). Professionals from high-income countries were more likely to advise screening than those from lower-middle-income countries, with odds ratios ranging from 2.9 to 12.3. Most health professionals reported recommending cancer screening for kidney transplant candidates and recipients. However, recommendations were influenced by costs and service delivery gaps within health systems.

## Graphical Abstract

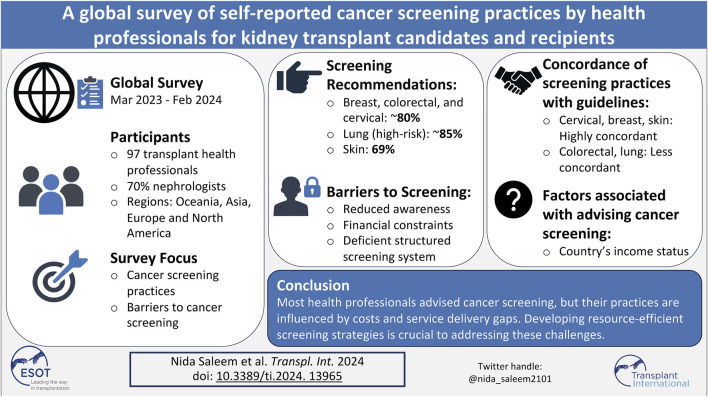

## Introduction

In kidney transplant recipients, cancer is a critically important outcome as it is one of the leading causes of death and the most feared complication of long-term immunosuppression [1, 2]. Compared to the age and sex-matched general population, the overall cancer incidence rates are at least double in kidney transplant recipients, with the increased risk varying depending on the cancer type. Kidney transplant recipients are particularly susceptible to virus-related and non-virus-related cancers, such as melanoma, non-melanoma skin cancers, cervical cancer, and post-transplant lymphoproliferative disease (PTLD) [3, 4]. The standardized incidence ratios (SIR) for these cancers range from 2.5 to 9.8 [5]. For non-immune-related cancers like colorectal and lung cancers, the risk is also elevated by approximately 2-3 times compared to the general population. Once cancer develops, the risk of death for kidney transplant recipients is about twice as high as for the general population [6]. This heightened mortality is due to the aggressiveness of cancers resulting from long-term immunosuppression and impaired immune surveillance. Additionally, the fear of acute allograft rejection from cancer-directed systemic therapies may jeopardize ongoing treatments for these high-risk patients.

To improve cancer-related outcomes, cancer screening plays a crucial role by facilitating early cancer detection and effective treatment before advanced-stage and aggressive diseases. Trial-based evidence in the general population has shown proven long-term mortality benefits with cancer screening, particularly concerning breast, colorectal, and cervical cancers [7–9]. Following recommendations from general population guidelines and evidence from observational studies, several transplant guidelines, like the Kidney Disease Improving Global Outcomes (KDIGO), the American Society of Transplantation (AST), and the European Best Practice Guidelines (EBPG), have recommended age-appropriate cancer screening for kidney transplant candidates and recipients [10]. However, prior research has indicated that guidelines are not often applied.

Despite recommendations by clinical practice guidelines, uptake of cancer screening remains low among transplant recipients [11, 12]. In Canada, less than 50% of women with kidney transplants participated in routine cervical and breast cancer screening, whereas over 70% of women without chronic kidney disease received regular Pap smears or human papillomavirus (HPV) tests and mammography [12]. Patients with kidney transplants may face many challenges, including concurrent comorbid conditions such as infections and cardiovascular diseases, limited access to preventive care, and prioritization of ongoing health issues, such as maintaining optimal allograft function, over other less imminent problems, such as cancer [13]. Similarly, a lack of health providers’ cancer screening recommendations and follow-up, limited knowledge, and health literacy may impact screening participation [14]. Delayed diagnosis and treatment may result in poorer outcomes.

One of the key elements for successful implementation involves identifying and understanding the potential barriers at the patient, provider, and organizational levels and devising strategies to address these barriers [15]. Many studies have emphasized the pivotal role that health professionals play in improving cancer screening participation for their patients, as they are a direct and trusted source of health information [16, 17]. In transplantation, health professionals’ knowledge, practices, and the challenges they encounter in clinical settings are unknown.

This study aimed to gain insights into the disparities and gaps in cancer screening implementation among transplant health providers by describing their global cancer screening practices for kidney transplant candidates and recipients, identifying barriers, and evaluating factors influencing their cancer screening behaviours.

## Materials and Methods

### Study Design

We formulated a questionnaire that assessed the cancer screening practices of health professionals working in nephrology, including nephrologists, nephrology trainees, transplant surgeons, nurses, and transplant coordinators, for kidney transplant candidates and recipients. After reviewing literature and cancer screening guidelines, the survey was developed with our patient partners (Consumer representatives at the Center for Kidney Research and members of key consumer groups; Better Evidence and Translation-Chronic Kidney Disease (BEAT-CKD) [18] and Centers of Research Excellence: Partnering with Patients with Chronic Kidney Disease (CRE-PACT) [18]) and piloted among fifteen experts from a local health district and a kidney research center in Sydney, Australia, to ensure its appropriateness, ease, and understandability. The survey was modified according to the suggestions of these research experts.

The survey contained three sections. The first included nine questions regarding the respondents’ demographic and professional details. The second section assessed their referral patterns and barriers that may influence the participants’ choices. Lastly, the third component included questions regarding their site-specific screening practices, including their advice regarding types of screening, modality, and frequency for both transplant candidates and recipients. The detailed survey is attached to the Supplementary Material (Supplementary Material S1).

Informed consent was obtained electronically from the survey participants. We then used adaptive questioning, a survey technique where survey questions are tailored based on the participants’ previous responses, to minimize the number of questions and enhance the relevance of the survey experience for each respondent [19]. Responders had the opportunity to check the completeness of their responses and review them using the back buttons. To prevent duplicative responses, the survey was distributed exclusively through unique invitation links.

### Participants

A closed web-based questionnaire was sent via email to all members of the Australia and New Zealand Society of Nephrology (ANZSN), Transplantation Society of Australia and New Zealand (TSANZ), BEAT-CKD, and The Transplantation Society (TTS) working network contact directory and through personal contacts from March 2023 to February 2024. Global health professionals currently working in nephrology, including nephrologists, nephrology professionals in training (trainee, resident, fellow), kidney transplant surgeons, nurses, and transplant coordinators, were invited to participate. After the initial post, one reminder email was sent to those who had yet to respond. All information on the questionnaire was de-identified to ensure confidentiality. No financial incentives were provided to the respondents. Ethics approval for this study was obtained from the University of Western Australia Human Ethics Committee (Approval Number: 2022/ET000790) following the guidelines set forth by the National Health and Medical Research Council (NHMRC). Reciprocal approval was granted by Flinders University’s Research Ethics and Compliance Office (Approval Number: 5966). We followed the Checklist for Reporting Results of Internet E-Surveys (CHERRIES) checklist to report this study [19].

### Statistical Analysis

Cancer screening practices of health professionals were analyzed using descriptive statistics. The proportions of participants who advised breast, colorectal, cervical, skin, lung, prostate and kidney cancer screening were estimated and graphically represented using clustered bar charts. Missing data was excluded while calculating these proportions.

Similarly, the proportion of site-specific cancer screening practices of transplant health professionals was described and compared with widely accepted transplant guidelines such as Kidney Disease: Improving Global Outcomes (KDIGO), American Society of Transplant (AST), Canadian Society of Transplantation and the Canadian Society of Nephrology (CST-CSN), European Best Practice Guidelines (EBPG), Kidney Health Australia-Caring for Australasians with Renal Impairment (KHA-CARI) and Renal Association (RA) Clinical Practice Guidelines. Specifically, we reviewed practices against the recommendations for breast, colorectal, cervical, skin, and lung cancer screening modalities, frequencies, and starting and stopping ages (Table 1). We noted the proportions of health professionals’ cancer screening responses and defined concordance between their screening practices and the guidelines as strong (>75%), moderate (50- ≤ 75%), weak (25- ≤ 50%), and very weak (≤25%).

**TABLE 1:** Clinical practice guidelines on cancer screening in kidney transplant recipients.

Guidelines	AST [20–22] (2000, 2009)^a^	CST-CSN [23, 24] (2010)^a^	KDIGO [10, 25, 26] (2009)^a^	RA [27, 28] (2017)^a^	KHA-CARI [29, 30] (2012)^a^	EBPG [31, 32] (2002)^a^
Breast cancer	Every 1–2 yearly mammography between 50 and 69 years	Every **2–3** yearly mammography between **50** to **74** years^b^	Annual mammography above **50** years^b^	Every **3** yearly mammography between **50** to **70** years^b^	Every **2** yearly mammography between **50** to **74** years^b^	Mammography between **45** to **74** years^b^
Cervical cancer	Annual PAP cytology between 18 to **65** years OR Every 3 yearly HPV testing^c^ [33, 34]	Annual PAP cytology between **25** to **69** years^b^	Every 3 yearly PAP cytology between **21** to **65** years OR Every **5** yearly **HPV** testing till **65** years^b^ [26]	Every **3** yearly PAP cytology between **25** to **49** years than **5** yearly till **65** years^b^	PAP cytology between **25** to **74** years OR Every 3 yearly HPV testing^c^ [35]	Annual PAP cytology between **25** to **65** years OR Every **5** yearly **HPV** testing between **30** to **65** years^b^ [32]
Colorectal cancer	Annual FIT^d^ OR Every 5 yearly sigmoidoscopy between 50 to **75** years	Every **2** yearly FIT^d^ OR Every **10** yearly sigmoidoscopy between **50** to **74** years^b^	Annual FIT^d^ OR Every **5–10** yearly sigmoidoscopy between **50** to **75** years Colonoscopy if FIT^d^ positive^b^	Every **2** yearly FIT^d^ between **50** to **74** years Colonoscopy if FIT^d^ positive^b^	Every **2** yearly FIT^d^ between **45** to **74** years^b^ Colonoscopy if FIT^d^ positive^b^	FIT^d^ between **50** to **74** years^b^ Colonoscopy if FIT^d^ positive^b^
Skin cancer	Monthly skin self-exam Annual physician exam	Skin self-exam Annual Specialist exam	Skin self-exam Annual Specialist exam	Biennial physician exam for 5 years post-transplant than annually	Skin self-exam Annual Specialist exam	—
Lung cancer	**Annual** CT-chest between **50** to **80** years	—	—	—	—	—

^a^ Bold indicates screening modalities, frequencies, starting and stopping ages following the KDIGO, transplant candidate guidelines [26] and current American [22], Canadian [24], United Kingdom [28], Australian [30], and European general population guidelines [32].

^b^ Cancer screening as per general population guidelines.

^c^ HPV, testing frequency based on American Society of Transplantation Infectious Disease guidelines and Australian Cancer Council guidelines.

^d^ Faecal Immunochemical test.

We used logistic regression modeling to determine the association between demographic and clinical factors and the willingness to recommend screening for breast, colorectal, and cervical cancers. We also included factors such as clinicians’ work experiences, their cancer screening system, and countries of practice, categorizing based on income status according to the World Bank classification [36]. An odds ratio (OR) of >1, with a 95% confidence interval, indicated a greater likelihood of cancer screening advice compared to the reference group. All statistical analyses were performed using SPSS [37].

## Results

**FIGURE 1:**
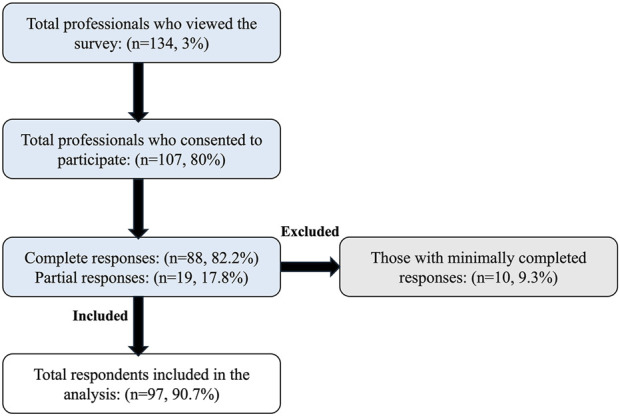
A flow diagram showing health professionals’ inclusion process.

Approximately 4500 health professionals working in the kidney transplant setting were invited to participate, with 134 viewing the survey (view rate 3%). Of the 134 survey viewers, 107 consented to participate (participation rate 80%). Among 107 participants, 88 completed the survey (completion rate 82%), while 19 (18%) provided partial responses. Of the 19 respondents who provided partial responses, 10 were excluded for completing less than 30% of the survey. As a result, data from 97 respondents were included in the final analysis, as illustrated in the flow diagram (Figure 1).

Table 2 shows the demographic characteristics of all respondents. About half of the responders were males (53%), in the age 31–40 years category (50%), and worked in Australia (56%). Most were nephrologists (70%), with clinical experience of less than 10 years (64%) and working in urban (83%) and transplant settings (59%). In addition to their clinical roles, many reported contributing to the formulation of clinical practice guidelines (42%), and some reported working as a policymaker (13%) and holding governmental/institutional funding in kidney research (9%).

**TABLE 2:** Demographic characteristics of the survey respondents (n = 97).

Characteristics	n	%
Gender Male Female Prefer not to say	51 45 1	53 46 1
Age groups (years) 18–30 31–40 41–50 51–60 61–70	7 48 18 17 7	7 50 19 18 7
Country of residence based on income status High-income countries Australia New Zealand USA Others^a^ Lower-middle income countries Pakistan Vietnam	54 5 5 8 24 1	56 5 5 8 25 1
Primary role Nephrologist Transplant nurse Nephrology trainee/resident Others	68 15 8 6	70 16 8 6
Work experience (years) <10 11–20 21–30 >30	62 18 12 5	64 19 12 5
Location of work Urban Rural and remote Both	80 10 7	83 10 7
Practice setting^b^ Transplant centre Private dialysis centre Public dialysis centre	53 22 30	59 24 33

^a^ Includes health professionals from the United Kingdom, Saudi Arabia, Canada, Germany.

^b^ Missing data for seven respondents (Percentage calculation excludes missing data).

### Self-Reported Frequency of Providing Cancer Screening Advice To Kidney Transplant Candidates and Recipients

Among 97 respondents, 92 (95%) reported recommending cancer screening for kidney transplant candidates. Eighty-two (85%) reported recommending breast cancer screening, 78 (81%) cervical cancer screening, 76 (79%) colorectal cancer screening, 66 (69%) skin cancer screening, and 51 (53%) lung cancer screening. Only 11 (12%) respondents would recommend prostate cancer screening, and four (4%) would recommend kidney cancer screening.

**FIGURE 2:**
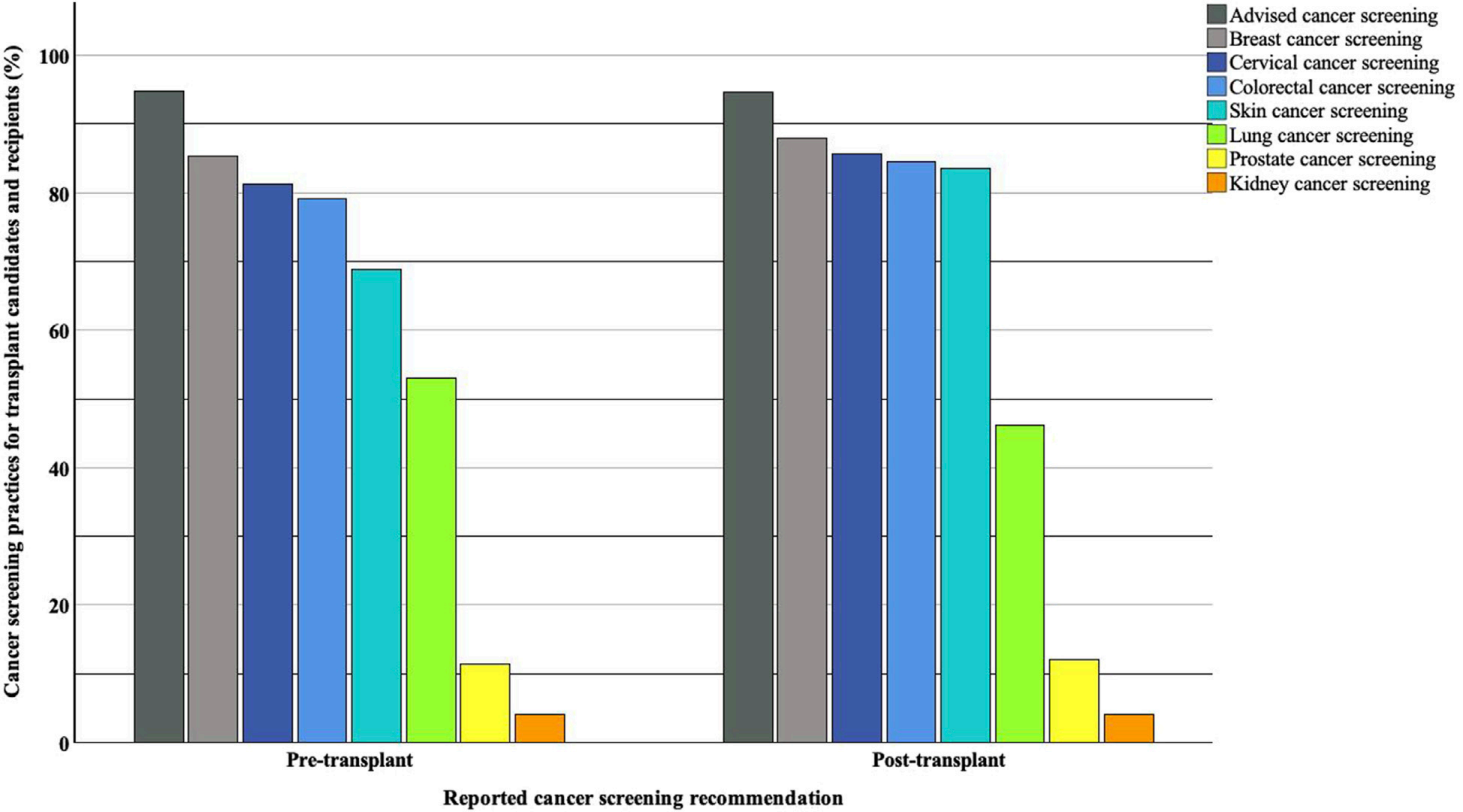
Reported cancer screening recommendations for kidney transplant candidates and recipients.

When asked about their practices for kidney transplant recipients, 90 out of 95 respondents reported recommending cancer screening (95%, with two missing responses). Similarly, out of 91 respondents (six missing), 80 (88%) reported recommending breast cancer screening, 78 (86%) cervical cancer screening, 77 (85%) colorectal cancer screening, 76 (84%) skin cancer screening, 42 (46%) lung cancer screening, 11 (12%) prostate cancer screening and 4 (4%) kidney cancer screening. The overall proportion of reported screening recommendations for all cancers was higher for kidney transplant recipients than candidates, except for lung cancer. A graphical representation of these cancer screening recommendations is shown in Figure 2.

### Site-Specific Cancer Screening Practices of Transplant Health Professionals

Table 3 shows site-specific cancer screening practices of transplant health professionals. Most respondents reported recommending biennial breast cancer screening (60%) by mammography (96%). Many respondents reported recommending a broad age range for breast cancer screening. Around 50% would initiate breast screening at the age of 40 and extend screening beyond 80. Some (34%) would continue breast cancer screening irrespective of age.

**TABLE 3:** Reported site-specific cancer screening practices among transplant health professionals.

Cancer screening practices		n	%
Breast cancer screening (n = 82)^a^			
Starting age, years	<40	13	16
	40	34	42
	50	30	37
	Unsure	5	6
Modality	Mammography	79	96
	Ultrasound	28	34
	MRI^b^ breast	9	11
	Breast self-examination	32	39
	Clinical breast examination	35	43
Frequency	Annually	21	26
	Biennially	49	60
	Others	6	7
	Unsure	6	7
Stopping age, years	>70	32	39
	>80	12	15
	Continue regardless of age	28	34
	Unsure	10	12
Male breast cancer screening	Yes	12	15
	No	52	63
	Unsure	18	22
Cervical cancer screening (n = 82)^a^			
Starting age, years	<18	2	2
	18–25	26	32
	When sexually active	49	60
	Unsure	5	6
Modality	Conventional cytology	46	56
	HPV^c^ testing	24	29
	Liquid-based cytology	8	10
	Unsure	4	5
Frequency	Annually	16	20
	Every 2–3 years	38	46
	Every 5 years	18	22
	Other	6	7
	Unsure	4	5
Stopping age, years	>70	39	48
	>80	3	4
	Continue regardless of age	23	28
	Unsure	17	21
Colorectal cancer screening (n = 82)^a^			
Starting age, years	<40	10	12
	40	12	15
	50	44	54
	60	7	9
	>60	1	1
	Unsure	8	10
Modality	Blood plasma test	3	4
	Colonoscopy	6	7
	CT^d^ colonoscopy (Virtual colonoscopy)	5	6
	FOBT^e^ (guaiac or immunohistochemical)	54	66
	Sigmoidoscopy (rigid or flexible)	7	9
	Stool DNA test (FIT^f^-DNA test)	3	4
	Unsure	4	5
Frequency	Annually	14	17
	Every 2–3 years	42	51
	Every 5 years	18	22
	Other	3	4
	Unsure	5	6
Stopping age, years	>70	26	32
	Continue regardless of age	43	52
	Unsure	13	16
Lung cancer screening (n = 82)^a^			
Average risk	Yes	38	46
	No	36	44
	Unsure	8	10
High risk^g^	Yes	70	85
	No	5	6
	Unsure	7	9
Starting age, years	<40	15	18
	40	13	16
	50	21	26
	60	3	4
	>60	1	1
	Unsure	29	35
Modality	Chest radiography	32	39
	Low-dose CT chest	33	40
	Other	1	1
	Unsure	16	20
Frequency	Annually	15	18
	Every 2–3 years	24	29
	Every 5 years	15	18
	Other	6	7
	Unsure	22	27
Stopping age, years	>70	19	23
	>80	7	9
	Continue regardless of age	30	37
	Unsure	26	32
Skin cancer screening (n = 75)^a^			
Modality	Full body skin check by dermatologist	39	52
	Full body skin check by GP^h^/non-skin specialist	27	36
	Skin self-check	8	11
	Unsure	1	1
Frequency-average risk	Annually	57	76
	Every 2–3 years	10	13
	Every 5 years	3	4
	Other	1	1
	Unsure	4	5
Frequency-high risk^i^	Every 3 months	11	15
	Every 6 months	37	49
	Annually	20	27
	Every 2-3 years	1	1
	Other	3	4
	Unsure	3	4

^a^ Sample size varies due to missing data (Percentage calculation excludes missing data).

^b^ Magnetic resonance imaging.

^c^ Human papillomavirus.

^d^ Computerised tomography.

^e^ Faecal occult blood test.

^f^ Faecal immunochemical test.

^g^ Defined as a current smoker or someone who has quit smoking in the past 15 years and has a smoking history of at least a 20-pack year.

^h^ General practitioner.

^i^ Defined as a personal or family history of skin cancer, a skin type more sensitive to UV damage, a history of severe sunburns, spending a lot of time outdoors, or using a solarium.

The majority of respondents reported recommending cervical cancer screening by conventional cytology (56%), commencing at 18–25 years or when sexually active (92%), and stopping at over 70 years (48%). In addition to cytological evaluation, some (29%) professionals also advised HPV-DNA testing. However, their reported cervical cancer screening frequency was less than guideline recommendations (46%).

For colorectal cancer screening, many respondents advised fecal immunochemical testing (FIT) (66%) commencing at the age of 50 years (54%), and around 50% would suggest less frequent screening (less than biennial screening), and the majority (52%) would advocate for ongoing screening regardless of age.

Approximately 46% of health professionals reported recommending lung cancer screening among average-risk kidney transplant candidates and recipients. The most common screening modality was low-dose computer tomography (CT) chest in high-risk transplant candidates and recipients, defined as current smokers or have quit smoking in the past 15 years, with a 20-pack year smoking history. Many health professionals expressed uncertainty regarding the commencement, frequency, and cessation of lung cancer screening.

Many professionals advised skin cancer screening using whole-body skin examinations conducted by a dermatologist (52%) or a non-skin specialist (36%). Reported screening intervals were typically annual (76%) for average risk and six-monthly (49%) for high-risk transplant candidates and recipients.

### Comparison of Transplant Health Professionals’ Responses With the Recommendations by Clinical Practice Guidelines

**FIGURE 3:**
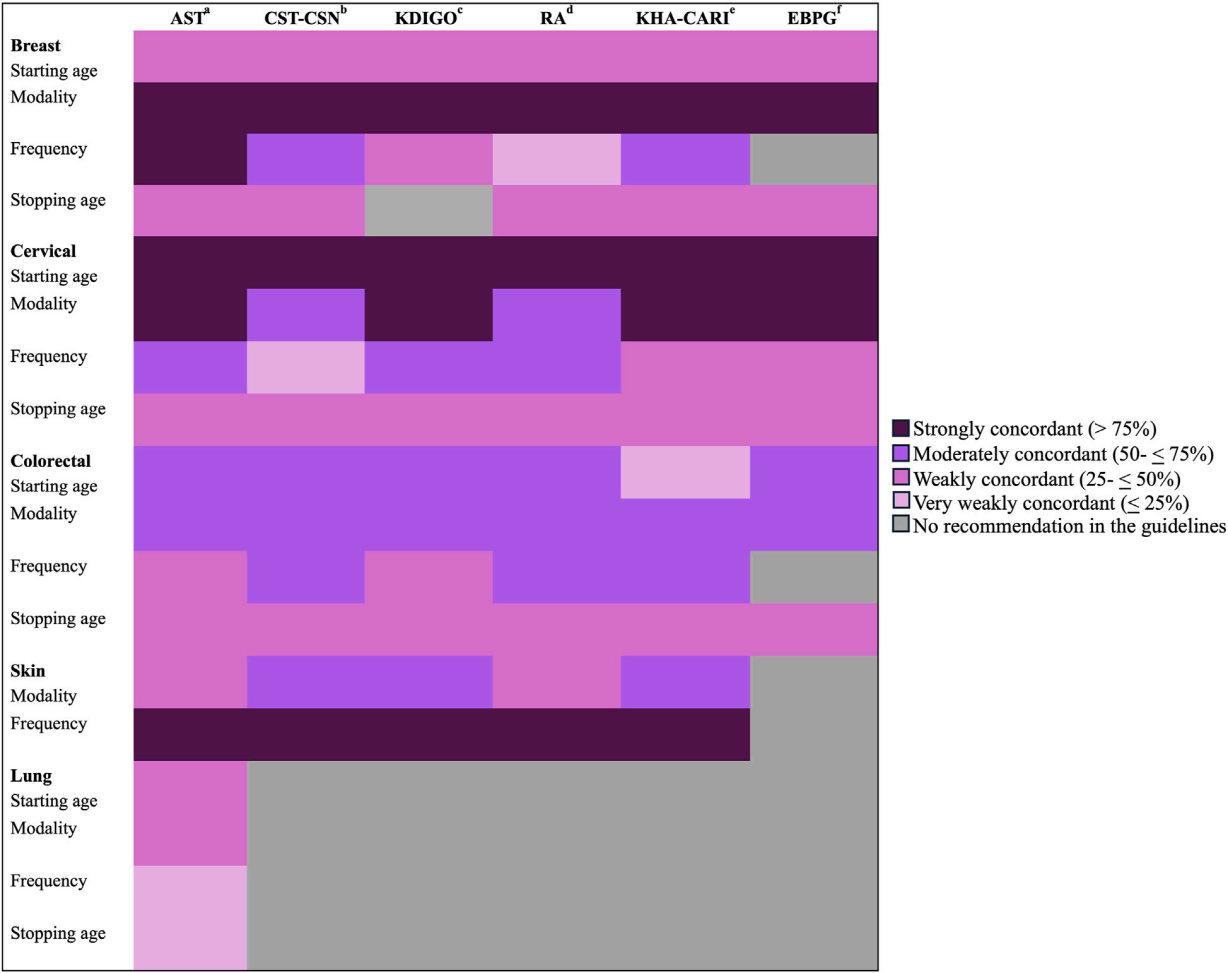
Concordance between reporting screening recommendations and clinical practice guidelines. a: American Society of Transplant, b: Canadian Society of Transplantation and the Canadian Society of Nephrology, c: Kidney Disease: Improving Global Outcomes, d: Renal Association Clinical Practice Guidelines, e: Kidney Health Australia-Caring for Australasians with Renal Impairment, f: European Best Practice Guidelines.

Figure 3 shows the concordance of reported screening practices for kidney transplant candidates and recipients with clinical practice guidelines. Cervical cancer screening reported practices were most concordant with international clinical practice guidelines, followed by breast and skin cancer screening practices.

Conversely, health professionals reported a lower level of conformity regarding their practices for colorectal and lung cancer screening compared to established clinical guidelines. Specifically, more than 50% of respondents favored continuing colorectal screening regardless of age. Also, only 40% advised CT-chest for lung cancer screening. Less than 20% of the participants reported recommending annual colorectal and lung cancer screenings, showing very weak concordance with many guidelines’ recommendations.

### Barriers Influencing Transplant Health Professionals’ Cancer Screening Recommendations

Financial constraints (35%), lack of patient awareness (28%), and the lack of specialized cancer screening units (28%) were frequently reported barriers to screening. Another prevailing barrier impacting their cancer screening advice was the lack of a structured screening system, especially in the post-transplant setting. While 76% of respondents indicated having a structured screening system for transplant candidates, the majority (51%) reported a lack of a structured screening system for transplant recipients in their clinical setting.

**FIGURE 4:**
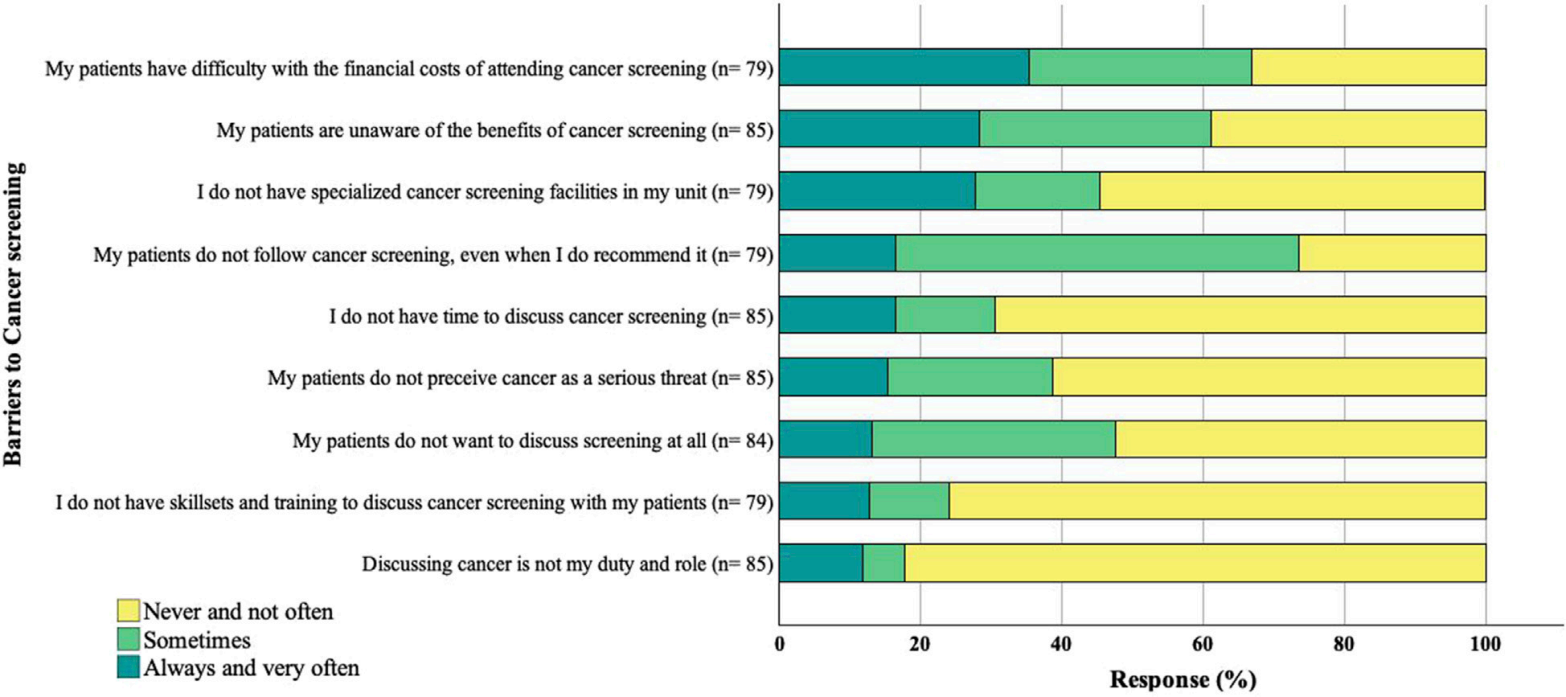
Reported barriers to cancer screening. The sample size for each response varies due to missing data.

In contrast, most health professionals denied having inadequate skills, training, and time as barriers to recommending screening. The majority acknowledged their duties to discuss cancer screening with transplant candidates and recipients (Figure 4).

### Factors Aligned With Reported Breast, Colorectal, and Cervical Cancer Screening Recommendations

As seen from Table 4, professionals from high-income countries (HIC), such as Australia, New Zealand, Canada, Germany, the United States, the United Kingdom, and Saudi Arabia, were more likely to recommend cancer screening in pre- and post-transplant settings than health professionals working in low-to middle-income countries (LMIC) such as Pakistan and Vietnam (odds ratios ranging between 2.9 and 12.3). Those with a working experience of greater than 10 years and those with a structured pre-transplant cancer screening system were more likely to advocate cancer screening for kidney transplant candidates, especially for cervical cancer (odd ratios of 5.9, CI: 1.3–27.3 and 9.2, CI: 2.2–38.3). However, these factors did not influence reported cancer screening recommendations for transplant recipients.

**TABLE 4:** Factors impacting transplant health professionals’ site-specific cancer screening practices in pre- and post-transplant settings^a^.

Factors^b^	p-value	OR^c^ (95% CI^d^)
For kidney transplant candidates		
Pre-transplant reported breast cancer screening recommendation		
Gender^e^ Male (n = 51) Female (n = 44)	0.38	1.0 0.6 (0.2–1.9)
Country of practice Lower-middle-income^f^ (n = 25) High income^g^ (n = 71)	**<0.001**	1.0 5.1 (1.6–16.7)
Work experience Less than 10 years (n = 61) Greater than 10 years (n = 35)	0.08	1.0 4.0 (0.9–19.2)
Have a structured cancer screening system^h^ No (n = 16) Yes (n = 65)	0.13	1.0 3.5 (0.7–17.7)
Pre-transplant reported cervical cancer screening recommendation		
Gender^e^ Male (n = 51) Female (n = 44)	0.17	1.0 0.5 (0.2–1.4)
Country of practice Lower-middle-income^f^ (n = 25) High income^g^ (n = 71)	**<0.001**	1.0 5.3 (1.8–15.6)
Work experience Less than 10 years (n = 61) Greater than 10 years (n = 35)	**0.02**	1.0 5.9 (1.3–27.3)
Have a structured cancer screening system^h^ No (n = 16) Yes (n = 65)	**0.00**	1.0 9.2 (2.2–38.3)
Pre-transplant reported colorectal cancer screening recommendation		
Gender^e^ Male (n = 51) Female (n = 44)	0.17	1.0 0.5 (0.2–1.4)
Country of practice Lower-middle-income^f^ (n = 25) High income^g^ (n = 71)	**<0.001**	1.0 7.3 (2.5–21.3)
Work experience Less than 10 years (n = 61) Greater than 10 years (n = 35)	0.09	1.0 2.8 (0.8–9.0)
Have a structured cancer screening system^h^ No (n = 16) Yes (n = 65)	0.21	1.0 2.4 (0.6–9.2)
For kidney transplant recipients		
Post-transplant reported breast cancer screening recommendation		
Gender^e^ Male (n = 49) Female (n = 41)	0.21	1.0 2.5 (0.6–10.0)
Country of practice Lower-middle-income^f^ (n = 23) High income^g^ (n = 68)	0.11	1.0 2.9 (0.8–10.5)
Work experience Less than 10 years (n = 58) Greater than 10 years (n = 33)	0.50	1.0 0.7 (0.2–2.3)
Have a structured cancer screening system^i^ No (n = 44) Yes (n = 28)	0.17	1.0 0.3 (0.1–1.7)
Post-transplant reported cervical cancer screening recommendation		
Gender^e^ Male (n = 49) Female (n = 41)	0.58	1.0 1.4 (0.4–4.7)
Country of practice Lower-middle-income^f^ (n = 23) High income^g^ (n = 68)	0.07	1.0 3.1 (0.9–10.4)
Work experience Less than 10 years (n = 58) Greater than 10 years (n = 33)	0.86	1.0 0.9 (0.3–3.0)
Have a structured cancer screening system^i^ No (n = 44) Yes (n = 28)	0.16	1.0 0.3 (0.1–1.5)
Post-transplant reported colorectal cancer screening recommendation		
Gender^e^ Male (n = 49) Female (n = 41)	0.83	1.0 1.1 (0.4–3.6)
Country of practice Lower-middle-income^f^ (n = 23) High income^g^ (n = 68)	**<0.001**	1.0 12.3 (3.3–45.3)
Work experience Less than 10 years (n = 58) Greater than 10 years (n = 33)	0.52	1.0 1.5 (0.4–5.3)
Have a structured cancer screening system^i^ No (n = 44) Yes (n = 28)	0.28	1.0 0.5 (0.1–1.9)

Bold values indicate significant p-values for factors.

^a^ Calculated through univariate logistic regression modelling.

^b^ Sample size varies due to missing data.

^c^ Odds ratio.

^d^ Confidence interval.

^e^ One health professional hasn’t mentioned gender.

^f^ Transplant health professionals from Pakistan and Vietnam.

^g^ Transplant health professionals working in Australia, New Zealand, the United States of America, the United Kingdom, Canada, Germany and Saudi Arabia.

^h^ Five professionals were unsure of the pre-transplant structured cancer screening system.

i 14 professionals were unsure of the post-transplant structured cancer screening system.

## Discussion

Consistent with clinical practice guidelines for cancer screening in kidney transplant candidates and recipients, our study found that most transplant health professionals reported recommending breast, colorectal, and cervical cancer screening to their patients. Transplant health professionals were more likely to recommend skin cancer screening for kidney transplant recipients than for candidates, while lung cancer screening was less frequently recommended, accompanied by a lot of reported uncertainties. Transplant health professionals proposed a broader age range for starting and stopping cancer screening compared to clinical practice transplant guidelines. Screening practices were influenced by factors such as cancer screening awareness among patients, the availability of health system resources, and the financial constraints faced by both patients and health facilities.

Studies to date have reported that transplant recipients can benefit from age-appropriate population-based screening practices, including breast, colorectal, and cervical cancers [38]. Similarly, our study findings showed cervical, breast, and skin cancer screening practices among health professionals were consistent with published guidelines. A higher conformity with cervical screening practices may be influenced by improved knowledge regarding test performance, the cost-benefit ratios of screening using HPV-DNA testing [39], especially self-testing, and access to updated cervical cancer screening transplant guidelines [33, 34], in contrast to most transplant guidelines published before 2012. Similarly, a high concordance with breast cancer screening guidelines was observed, likely attributed to greater awareness and robust trial-based evidence showing mortality benefits in the general population [40]. Likewise, a greater alignment of skin cancer screening practices and guidelines may be due to the uniform advocacy of skin cancer screening by transplant health professionals. This advocacy is driven by heightened awareness of the disease burden and higher cumulative incidence of skin cancers compared to the age and sex-matched general population [3, 41].

Several inconsistencies were evident between the self-reported screening practices of transplant health professionals and the recommended guidelines. For instance, most transplant health professionals suggested broader age ranges for screening, particularly for colorectal and breast cancers. There were also discrepancies across the recommended frequency for colorectal screening and uncertainties regarding the frequency and timing of lung cancer screening. Clinicians would also recommend less frequent screening for colorectal and lung cancers. Only 29% would recommend HPV-DNA testing for cervical cancer screening in addition to PAP cytology, and 40% would use low-dose CT chest for lung cancer screening for high-risk individuals.

There are likely to be many reasons for the observed discordance. Health professionals may prioritize other competing health issues experienced by transplant recipients, such as maintaining optimal graft function, over future events, such as cancer, that are not immediately imminent [42, 43]. Similarly, the screening practices among health professionals are also highly variable. These practices are largely driven by their patients’ cancer risk, expected survival, preferences, comorbidities and ongoing treatment burden, leading to inconsistent screening advice [16, 43]. Furthermore, the lack of uniformity between cancer screening guideline recommendations likely contributes to their inconsistent screening advice. The recommendations to target broader age ranges for cancer screening may be influenced by the growing evidence suggesting heightened cancer risk and a more aggressive cancer course in younger transplant recipients [44]. Current clinical guidelines suggest primary HPV testing every 3–5 years for transplant recipients compared to conventional cytology screening biennially [45]. However, the transition to HPV testing from conventional cytology has not been universally adopted by LMIC due to various barriers, including a lack of infrastructure for high-complex molecular testing and equipment, limited screening system, laboratory capacity, skilled expertise, and human resources, and centralization of laboratories [45, 46]. While there is now robust trial-based evidence to suggest lung cancer screening using low-dose CT among high-risk individuals reduces the risk of lung cancer-related death by 20%–24% compared to no screening [47, 48], population-based screening programs have not commenced in many countries. For instance, in Australia, the proposed launch date for the National lung cancer screening program is in July 2025 [49]. Similarly, in the United Kingdom (UK), the National Health Service will roll out the program in 2025, with full coverage anticipated in 2030 [50].

Other factors, such as economic deprivation, inadequate healthcare funding, infrastructure, and resources, may also impact cancer screening decisions [12, 51–53]. A robust, well-organized, well-governed, publicly funded population-based screening program is needed to maximize uptake and participation in cancer screening. However, these systems are lacking in many LMICs [52], as reported by health professionals residing in countries such as Pakistan and Vietnam in our study. In addition to reliable health investments, education about the potential benefits and harms of routine screening and recommendations is crucial. Misinformation and the lack of awareness among patients and clinicians may lead to under-utilization and inappropriate screening [52, 54]. Prior research has indicated many transplant recipients underestimated the importance of cancer screening [55, 56].

Other strategies that may facilitate the successful implementation of cancer screening for transplant recipients within both income settings include involving primary care physicians in screening advice [17], ensuring a continuum of care at transplant centers [17], adopting an individualized risk-based approach to screening, and promoting shared decision-making by considering various factors including patients’ life expectancy, graft health, comorbidities, and the recipients’ age in cancer screening decisions [17, 43, 57]. Also, there is a need for regularly updating society-based guidelines, ensuring the recommendations remain aligned with the most current evidence. Furthermore, incorporating and educating about self-testing for HPV-DNA and FIT may improve screening compliance and limit the burden on providers and health resources [52, 58]. Other interventions like mobile screening to mitigate travel costs and employing patient navigators may improve screening adherence [52, 59].

This study has several limitations. Despite developing a well-designed survey, conducting thorough pilot testing, and sending reminder emails [60], limited survey view rates remain a key limitation, likely due to professionals’ lack of interest or time for cancer screening [61], impacting the generalizability of our study findings. However, we have not explicitly investigated the factors that may contribute to these limited survey view rates. Also, most of the respondents were nephrologists (70%) with less than 10 years of work experience (63%) and primarily practicing in urban settings (80%), which may not fully reflect the cancer screening practices of more experienced transplant health professionals from rural and remote settings. While we attempted to sample participants from the relevant global transplant societies, we do not have representation from countries in Africa and other parts of Asia (including India and China). This study relied on self-reported data for assessing the cancer screening practices of health professionals, potentially introducing a reporting bias by overestimating their inclination towards cancer screening and underestimating their actual screening behaviours. This study, however, has several strengths. The survey was distributed globally to health professionals working in the field of post and pre-transplant care. The self-reported survey approach allowed us to gain insights into their perceived barriers to screening, which would not be possible by merely observing screening practices. Prior to dissemination, the survey was extensively reviewed by consumer representatives and pilot-tested among clinicians, patients, and caregivers. The survey was conducted via a secured online portal, ensuring the confidentiality and anonymity of the respondents.

In conclusion, our study has provided an overview of the key factors influencing cancer screening practices among transplant health professionals. Most respondents acknowledged the importance of screening among at-risk individuals and recognized their pivotal role in providing screening advice. However, the lack of resources and inadequate cancer screening systems significantly impacted their screening decisions, highlighting the need for attention in these areas. Implementing the widely accepted screening guidelines’ recommendations developed in high-income countries may not be feasible in low-resource settings, and there is an urgent need to implement cancer screening programs desired for low-income transplant settings. Future studies are imperative to develop and evaluate cost-effective screening strategies in LMIC, ensuring equitable and accessible post-transplant care for all.

## Data Availability

The raw data supporting the conclusion of this article will be made available by the corresponding author upon request.

## Abbreviations

ANZSN, Australian and New Zealand Society of Nephrology; AST, American Society of Transplant; BEAT-CKD, Better Evidence and Translation-Chronic Kidney Disease; CI, Confidence Interval; CRE-PACT, Centres of Research Excellence: Partnering with Patients with Chronic Kidney Disease; CST-CSN, Canadian Society of Transplantation and the Canadian Society of Nephrology; CT, Computed tomography; DNA, Deoxyribonucleic acid; EBPG, European Best Practice Guidelines; FIT, Faecal Immunohistochemical Test; FOBT, Faecal Occult Blood test; GP, General practitioner; HPV, Human Papillomavirus; KDIGO, Kidney Disease: Improving Global Outcomes; KHA-CARI, Kidney Health Australia-Caring for Australasians with Renal Impairment; MRI, Magnetic resonance imaging; OR, Odds Ratio; PTLD, Post-Transplant Lymphoproliferative Disorder; RA, Renal Association; SIR, Standardised Incidence Ratio; SONG, Standardised Outcomes in Nephrology; TSANZ, Transplantation Society of Australia and New Zealand; TTS, The Transplantation Society; UK, United Kingdom; USA, United States of America.
