# Supplementary material for: A Global Survey of Self-Reported Cancer Screening Practices by Health Professionals for Kidney Transplant Candidates and Recipients
Source: Transpl Int. 2025 Jan 20;37:13965. doi: 10.3389/ti.2024.13965 (PMC11788010; doi:10.3389/ti.2024.13965)
Supplement: Supplementary file 1 [file DataSheet1.pdf]

Flinders University

Supplementary file:

Project title

Global survey of health professional's cancer screening recommendations and practices for kidney transplant candidates and recipients

Consent

Online Survey Participant Information

You are invited to participate in a project of cancer screening recommendation for kidney transplant candidates and kidney transplant recipients. Your opinions are valuable and important to better understand the utilization of cancer screening tests in routine clinical settings. The information will also provide valuable information for appropriate implementation of cancer screening practices in caring for kidney transplant candidates and transplant recipients.

What does participation involve?

You will be asked to complete a 20-30-minute survey to evaluate your knowledge, attitudes, barriers, and practices related to routine cancer screening in your clinical settings. The survey is designed to accommodate a wide range of practice settings and transplant health professionals, you may find some of the questions do not necessarily apply to you. In those situations, we still encourage you to complete the survey. All participants will receive a copy of the results. For more information, please click on the link below.

Uwa survey picf cancer screening.

Consent Statement

I have read the information provided and any questions I have asked have been answered to my satisfaction. I agree to participate in this research project, realizing that I may withdraw at any time without reason and without prejudice. I understand that all identifiable information that I provide is treated as confidential and will not be released by the investigator in any form that may identify me unless I have consented to this. The only exception to this principle of confidentiality is if this information is required by law to be released. The study has no information about the identity of the participants.

I consent to participate in this research project

○ Yes
○ No

Introduction:

Introduction:

This survey will ask about your attitudes and practices relating to cancer screening in kidney transplant candidates and recipients.

In this survey, we define cancer screening as the use of a test intended to identify transplant candidates and recipients who are at increased risk of developing cancer before clinical detection or incidental discovery. Patients with abnormal screening test results will be evaluated for further investigations to identify those with cancers. Cancer screening is used in patients without any signs or symptoms of possible cancer.

There are three parts in this survey:

Part A – Demographic details

Part B – General clinical aspects of cancer screening

Part C – Individual screening practices for breast, cervical, colorectal, skin and lung cancers

Your participation is voluntary, and you may exit the survey at any time.

Please complete these questions on your own. By clicking “submit” at the end of the survey, you are consenting for your data to be used for research purposes. Responses will be anonymous.

Declaration

Please confirm if you are a kidney transplant health professional (e.g. doctors, nurse, allied health, patient coordinator etc):

- ◯ Yes (please go to the next question)
- ◯ No (thank you, this survey does not apply to you)

Part A - Demographics

Part A - Demographics

1. What is your gender?

- ○ Male
- ○ Female
- ○ Other
- ○ Prefer not to say

2. What is your age group (in years)?

- ○ 18-30
- ○ 31-40
- ○ 41-50
- ○ 51-60
- ○ 61-70
- ○ 71-80
- ○ 81 and over

3. In which country do you currently live?

4. In which city do you currently live?

5. What is your primary role?

- Nephrologist
- Surgeon
- General practitioner
- Nurse
- Psychologist
- Dietitian
- Pharmacist
- Transplant coordinator
- Social worker
- Nephrologist in training (trainee, resident, fellow)
- Physician of other specialty
- Other (please specify)

6. How long have you worked in kidney transplantation (years)?

- ○ <10
- ○ 11 to 20
- ○ 21 to 30
- ○ >30

7. Is your practice in:

- ☐ Urban
- ☐ Rural and remote settings

8. Is your practice in a:

- ☐ Transplanting centre
- ☐ Private nephrology and dialysis centre
- ☐ Public nephrology and dialysis centre
- ☐ [ ] Others – please list

9. Please indicate if you have a role in any of the following?

- ☐ Government, policy maker

- [ ] Clinical practice guidelines
- [ ] Funding (government, institution, charity)
- [ ] Other (please specify)

Part B - Clinical aspects of cancer screening

Part B - Clinical aspects of cancer screening

1. Do you recommend cancer screening prior to transplantation?

- ◯ Yes
- ◯ No – Please specify the reason.

2. What types of cancer screening do you recommend for transplant candidates before wait-listing and transplantation? (please tick all that apply)

- [ ] Colorectal
- [ ] Breast
- [ ] Cervical
- [ ] Lung

- [ ] Skin
- [ ] Others – please specify

3. Do you recommend cancer screening for transplant recipients?

- ( ) Yes
- ( ) No – Please specify the reason.

4. What types of cancer screening would you recommend for transplant recipients? (please tick all that apply)

- [ ] Colorectal
- [ ] Breast
- [ ] Cervical
- [ ] Lung
- [ ] Skin
- [ ] Others – Please specify

5. Which cancer screening guideline do you follow?

- ☐ KDIGO guidelines
- ☐ General population guidelines
- ☐ Local transplant guidelines
- ☐ Other – Which ones? ________
- ☐ None – Why? ________

6. In your practice/unit, how influential are the current cancer screening guidelines for transplant candidates and recipients?

- ○ Very influential
- ○ Somewhat influential
- ○ Not influential
- ○ Not applicable/familiar with

7. In your practice/unit, do you have a structured system that refers patients to routine cancer screening before listing and transplantation?

- ○ Yes
- ○ No
- ○ Unsure

8. In your practice/unit, do you have a structured system that refers patients to routine cancer screening after transplantation?

- ◯ Yes
- ◯ No
- ◯ Unsure

9. How often do you encounter the following barriers to screening in your practice/unit?

	Always	Very often	Sometimes	Not often	Never
i) My patients do not want to discuss screening at all.	◯	◯	◯	◯	◯
ii) I do not have specialized cancer screening facilities in my unit.	◯	◯	◯	◯	◯
iii) My patients are unaware of the benefits of cancer screening.	◯	◯	◯	◯	◯
iv) My patients do not perceive cancer as a serious health threat.	◯	◯	◯	◯	◯
v) Discussing cancer screening is not my duty and role.	◯	◯	◯	◯	◯
vi) I do not have time to discuss cancer screening.	◯	◯	◯	◯	◯
vii) My patients have difficulty with the financial costs of attending cancer screening.	◯	◯	◯	◯	◯

	Always	Very often	Sometimes	Not often	Never
viii) I do not have the skillsets and training to discuss cancer screening with my patients.	○	○	○	○	○
ix) My patients do not follow through with the screening test, even when I do recommend it.	○	○	○	○	○

x) Others – please list.

Breast Cancer

Part C – Cancer specific screening practices

Breast Cancer

10. What is your recommended starting age for breast cancer screening among your average-risk female transplant recipients and transplant candidates?

- ○ Less than 40 years
- ○ 40 years
- ○ 50 years

○ 60 years
○ Older than 60 years
○ Unsure

11. What is your recommended stopping age for breast cancer screening among your average-risk female transplant recipients and transplant candidates?

○ > 70 years
○ > 80 years
○ Continue regardless of age
○ Unsure

12. Would you recommend breast cancer screening for male transplant candidates and recipients?

○ Yes
○ No
○ Unsure

13. What is your recommended screening modality for breast cancer? (please tick all that apply)

☐ Clinical breast exam (performed by practitioner)
☐ Breast self-exam (performed by patient)

☐ Mammography
☐ Magnetic resonance imaging (MRI)
☐ Ultrasound
☐ [ ] Others – please specify
☐ Unsure

14. What is your recommended frequency for breast cancer screening?

○ Annually
○ Biennially
○ [ ] Others – please specify
○ Unsure

Cervical Cancer

Cervical Cancer

15. What is your recommended starting age for cervical cancer screening amongst your average-risk female transplant recipients and transplant candidates?

○ < 18 years

- ○ 18-25 years
- ○ When sexually active
- ○ Unsure

16. What is your recommended stopping age for cervical cancer screening amongst your average-risk female transplant recipients and transplant candidates?

- ○ > 70 years
- ○ > 80 years
- ○ Continue regardless of age
- ○ Unsure

17. What is your recommended screening modality for cervical cancer?

- ○ Liquid-based (For example, Thin Prep)
- ○ Conventional cytology (For example, smear on a slide – Pap test)
- ○ Human Papillomavirus-based testing
- ○ [ ] Other – please specify
- ○ Unsure

18. What is your recommended frequency for cervical cancer screening?

- ○ Annually
- ○ Every 2-3 years
- ○ Every 5 years
- ○ [ ] Other – please specify
- ○ Unsure

Colorectal Cancer

Colorectal cancer

19. What is your recommended starting age for colorectal cancer screening amongst your average-risk transplant recipients and transplant candidates?

- ○ Less than 40 years
- ○ 40 years
- ○ 50 years
- ○ 60 years
- ○ Older than 60 years
- ○ Unsure

20. What is your recommended stopping age for colorectal cancer screening amongst your average-risk transplant recipients and transplant candidates?

- ○ > 70 years
- ○ Continue regardless of age
- ○ Unsure
- ○ [ ] Other – please specify

21. What is your recommended screening modality for colorectal cancer?

- ○ Fecal occult blood test (For example, guiac or immunochemical FOBT)
- ○ Stool DNA test (For example, FIT-DNA test)
- ○ Sigmoidoscopy (either flexible or rigid)
- ○ CT-colonoscopy (Virtual colonoscopy)
- ○ Blood plasma test (For example, to detect cancer biomarkers)
- ○ [ ] Other – please specify
- ○ Unsure

22. What is your recommended frequency for colorectal cancer screening?

- ○ Annually

○ Every 2-3 years
○ Every 5 years
○ [   ] Other – please specify
○ Unsure

Lung Cancer

Lung cancer

23. Would you recommend lung cancer screening in your average-risk transplant candidate and recipients?

○ Yes
○ No
○ Unsure

24. Would you recommend lung cancer screening in your higher-risk transplant candidate and recipients?

(High risk defined as: currently smoke or have quit in the past 15 years, and have at least a 20 pack-year smoking history)

○ Yes
○ No
○ Unsure

25. What is your recommended starting age for lung cancer screening among your transplant recipients and transplant candidates?

- ○ Less than 40 years
- ○ 40 years
- ○ 50 years
- ○ 60 years
- ○ Older than 60 years
- ○ Unsure

26. What is your recommended stopping age for lung cancer screening among your transplant recipients and transplant candidates?

- ○ > 70 years
- ○ > 80 years
- ○ Continue regardless of age
- ○ Unsure

27. What is your recommended screening modality for lung cancer?

- ○ Chest radiography

- ○ Low dose chest computerized tomography
- ○ [ ] Other – please specify
- ○ Unsure

28. What is your recommended frequency for lung cancer screening?

- ○ Annually
- ○ Every 2-3 years
- ○ Every 5 years
- ○ [ ] Other – please specify
- ○ Unsure

Skin Cancer

Skin cancer

29. Would you recommend skin cancer screening in all transplant candidates and recipients?

- ○ Yes
- ○ No
- ○ Unsure

30. What is your recommended screening modality for skin cancer?

- ○ Self-check
- ○ Full-body skin check conducted by dermatologist
- ○ Full-body skin check conducted by general practitioner and/or other non-skin specialist
- ○ [ ] Other – please specify
- ○ Unsure

31. What is your recommended frequency for skin cancer screening in average risk transplant candidates and recipients?

- ○ Annually
- ○ Every 2-3 years
- ○ Every 5 years
- ○ [ ] Other – please specify
- ○ Unsure

32. What is your recommended frequency for skin cancer screening in high-risk transplant candidate and

recipients?
(High risk defined as: personal or family history of skin cancer, a skin type that is more sensitive to UV damage, history of bad sunburns, spent a lot of time outdoors, have used solarium)

- ○ Every 3 months
- ○ Every 6 months
- ○ Annually
- ○ Every 2-3 years
- ○ [ ] Other – please specify
- ○ Unsure

Your valuable contribution to this is greatly appreciated. To submit your responses, please press the forward button.
